# Supplementary figures and images for: Experimentally-constrained biophysical models of tonic and burst firing modes in thalamocortical neurons
Source: PLoS Comput Biol. 2019 May 16;15(5):e1006753. doi: 10.1371/journal.pcbi.1006753 (PMC6541309; doi:10.1371/journal.pcbi.1006753)

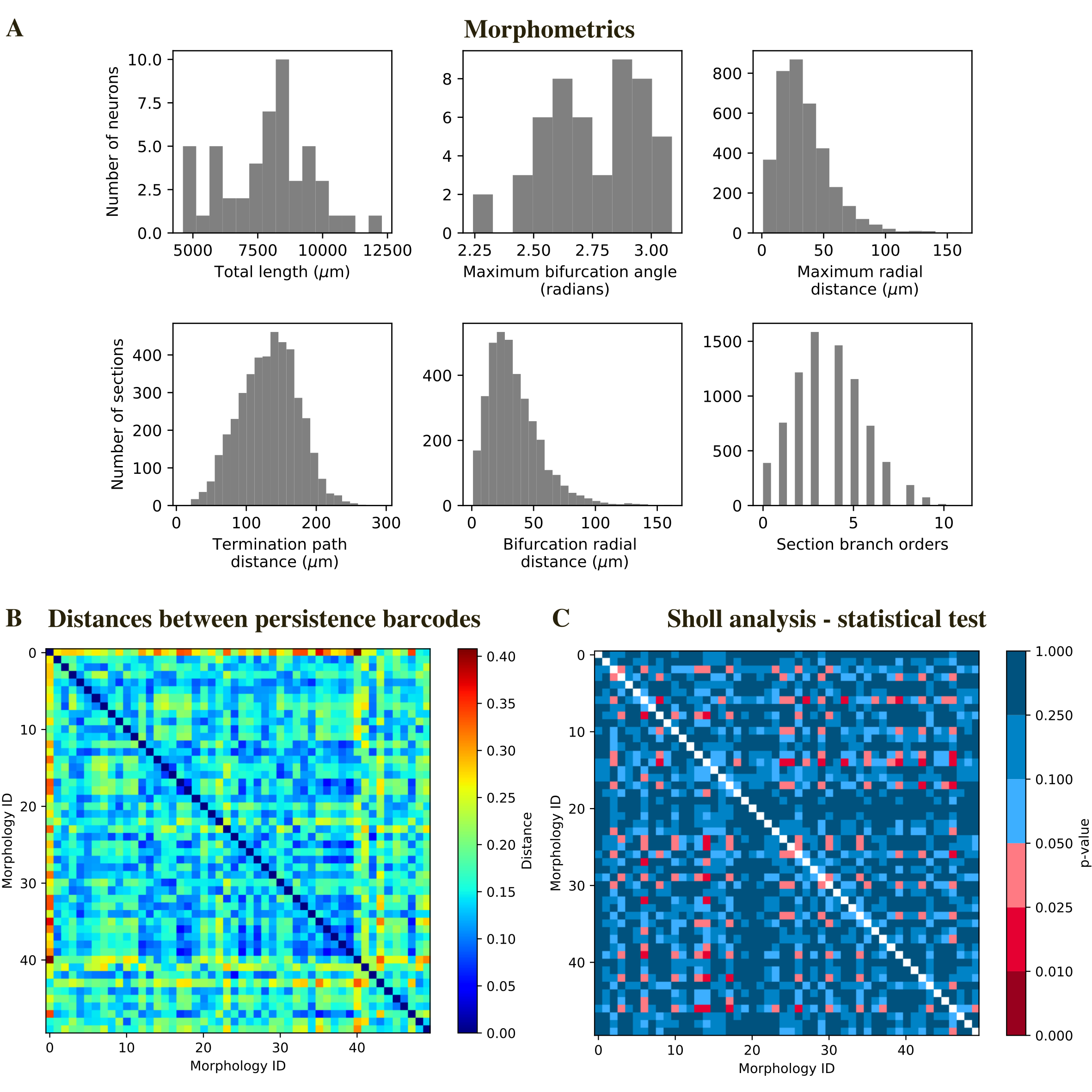

Supplement: S1 Fig — (A) Morphometrics of the thalamocortical (TC) morphological type. Each histogram shows basic morphometrics at the level of the neuron (first row) or at the level of dendritic trees (second row). (B) Distance matrix between persistence barcodes for all TC morphologies. Related to Fig 2A). (C) P-values for the k-samples Anderson-Darling statistics. It tests the null hypothesis that the Sholl profiles of each pair of morphologies are drawn from the same population. The p-values are not corrected for multiple comparisons and show that we cannot reject the null hypothesis for most of the morphology pairs (at 0.05 significance level). Related to Fig 2B. (TIF) [file pcbi.1006753.s001.tif]

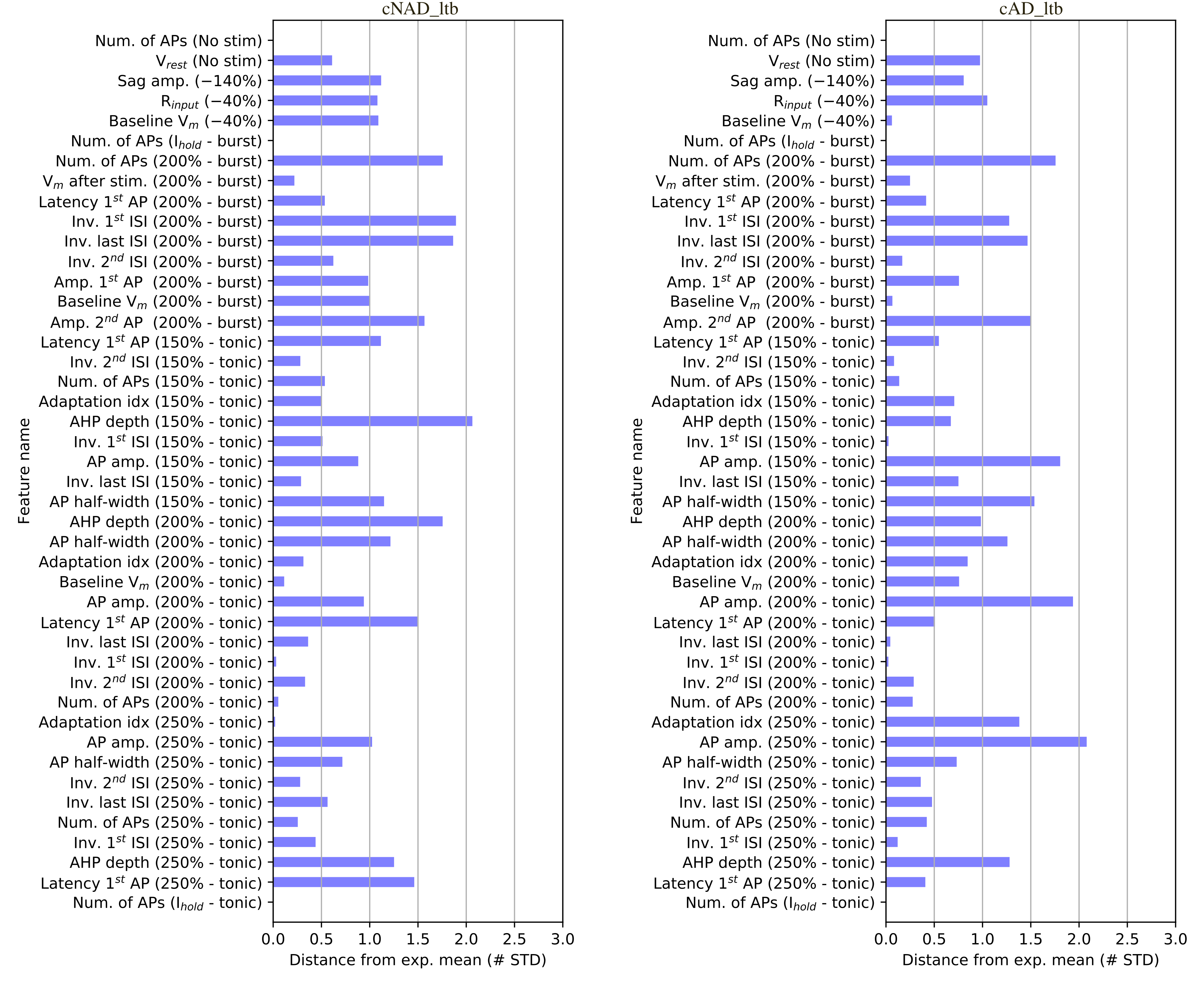

Supplement: S2 Fig — Detailed view of all feature errors for two optimized models; where the error bar is missing the error value is 0. (TIF) [file pcbi.1006753.s002.tif]

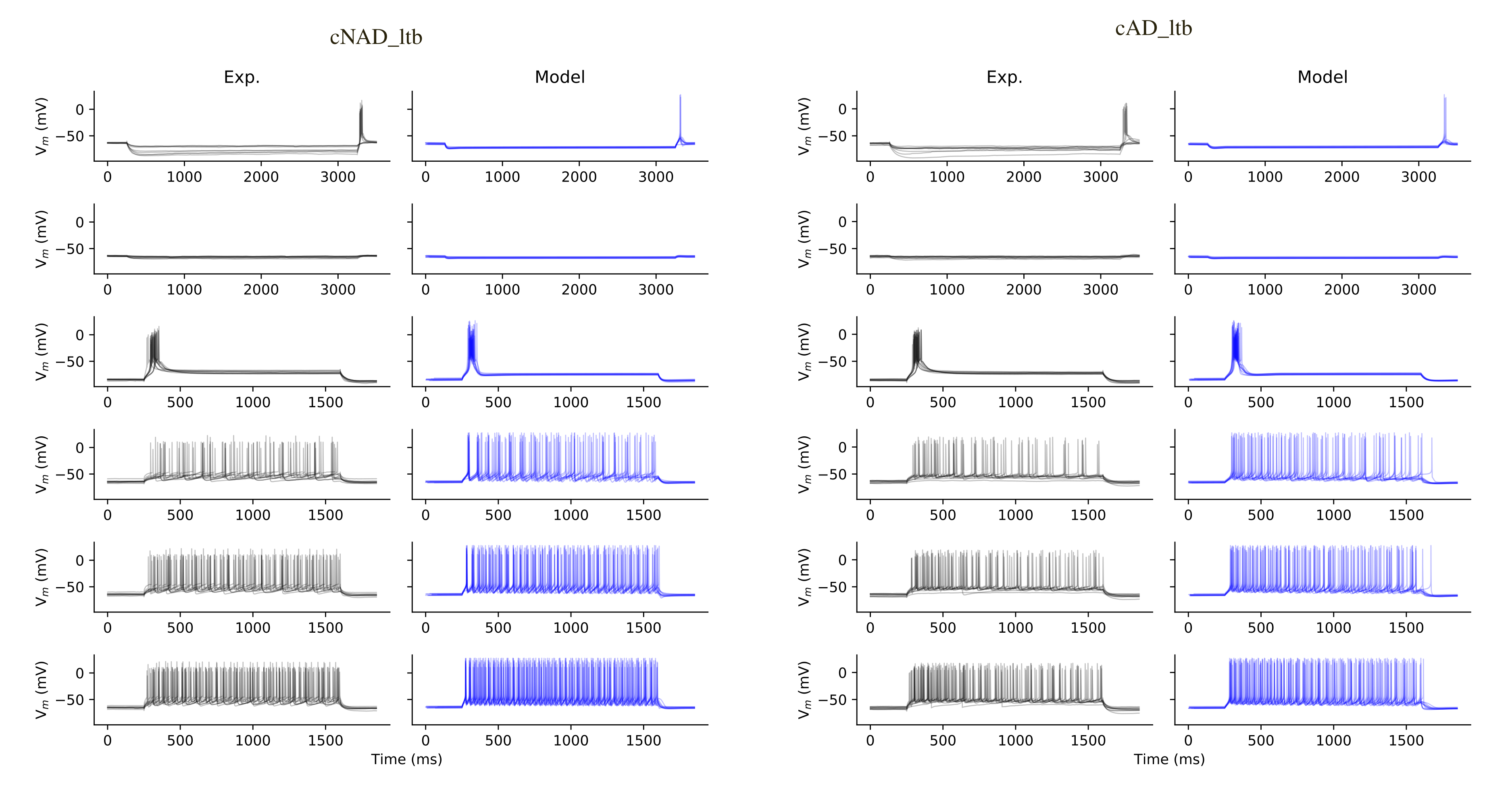

Supplement: S3 Fig — Voltage traces from a sample of 10 different experimental cells and models, corresponding to the features shown in Fig 5C. (TIF) [file pcbi.1006753.s003.tif]

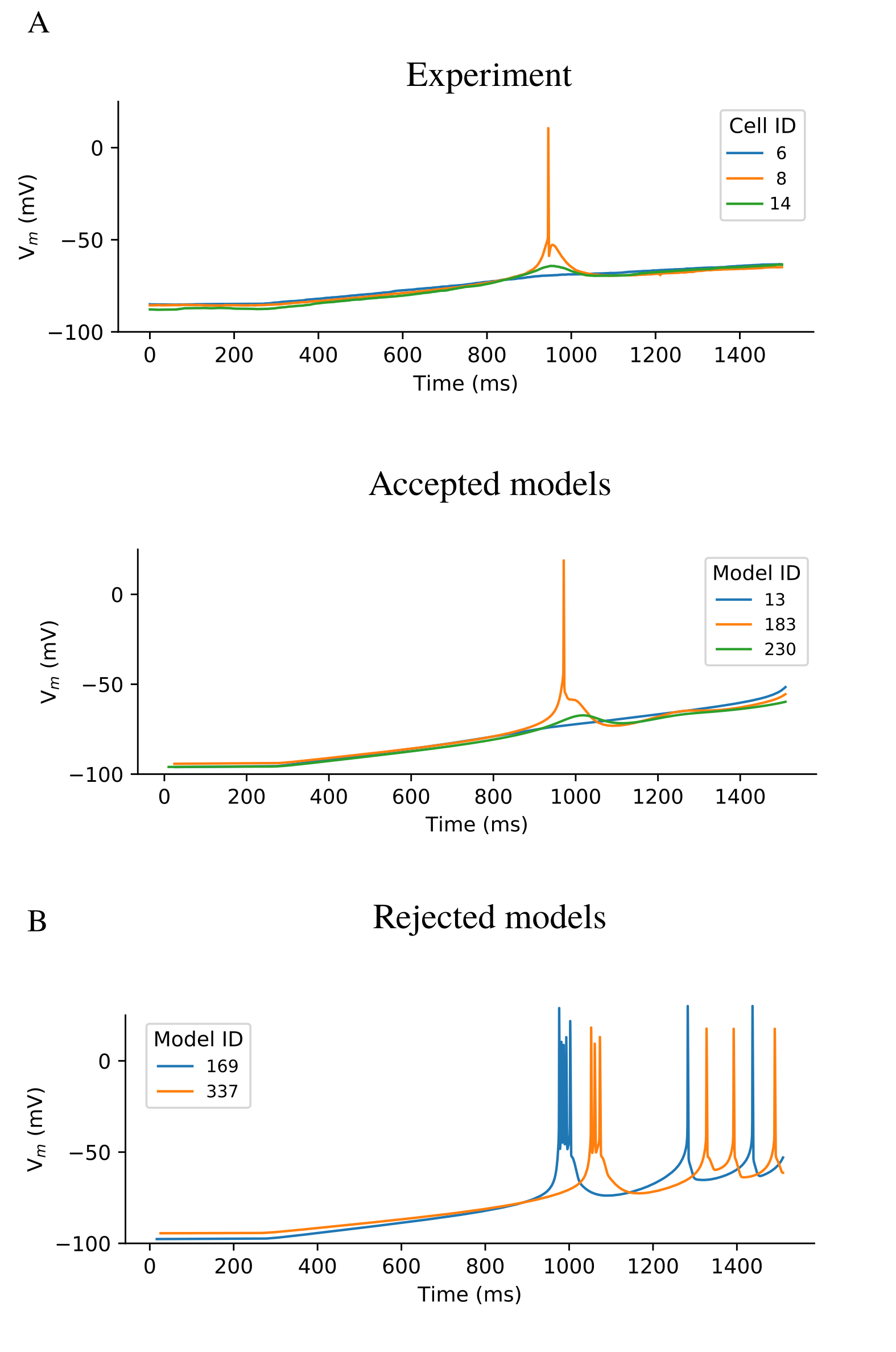

Supplement: S4 Fig — (A) Experimental cells and models show three different types of behavior, when recorded in burst mode: no firing (green), small low threshold spike (blue), low-threshold burst (low-threshold spike crowned by sodium spikes, orange). (B) Example of models that tend to have repetitive bursting or spiking behavior. (TIF) [file pcbi.1006753.s004.tif]
